# TP53INP2 knockdown inhibits inflammatory response and apoptosis after spinal cord injury

**DOI:** 10.1002/iid3.1256

**Published:** 2024-04-23

**Authors:** Penghao Sun, Jinchuan Chen, Rujie Qin

**Affiliations:** ^1^ Department of Spine Surgery The First People's Hospital of Lianyungang Lianyungang China

**Keywords:** inflammatory response, neuronal apoptosis, spinal cord injury, TP53INP2

## Abstract

**Background:**

Spinal cord injury (SCI) is a traumatic neurological disorder with limited therapeutic options. Tumor protein p53‐inducible nuclear protein 2 (TP53INP2) is involved in the occurrence and development of various diseases, and it may play a role during SCI via affecting inflammation and neuronal apoptosis. This study investigated the associated roles and mechanisms of TP53INP2 in SCI.

**Methods:**

Mouse and lipopolysaccharide (LPS)‐induced SCI BV‐2 cell models were constructed to explore the role of TP53INP2 in SCI and the associated mechanisms. Histopathological evaluation of spinal cord tissue was detected by hematoxylin and eosin staining. The Basso, Beattie, and Bresnahan score was used to measure the motor function of the mice, while the spinal cord water content was used to assess spinal cord edema. The expression of TP53INP2 was measured using RT‐qPCR. In addition, inflammatory factors in the spinal cord tissue of SCI mice and LPS‐treated BV‐2 cells were measured using enzyme‐linked immunosorbent assay. Apoptosis and related protein expression levels were detected by flow cytometry and western blot analysis, respectively.

**Results:**

TP53INP2 levels increased in SCI mice and LPS‐treated BV‐2 cells. The results of in vivo and in vitro experiments showed that TP53INP2 knockdown inhibited the inflammatory response and neuronal apoptosis in mouse spinal cord tissue or LPS‐induced BV‐2 cells.

**Conclusions:**

After spinal cord injury, TP53INP2 was upregulated, and TP53INP2 knockdown inhibited the inflammatory response and apoptosis.

## Graphical abstract

The present study investigated the roles and mechanisms of TP53INP2 in spinal cord injury (SCI) rat model and in vitro model, and its mechanism. Results indicated that after spinal cord injury, TP53INP2 knockdown inhibits inflammatory response and apoptosis. TP53INP2 may be a potentially effective therapeutic target for SCI.

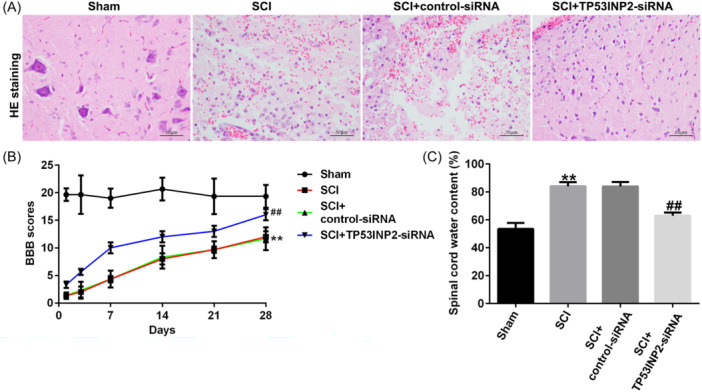

## 1 INTRODUCTION

Spinal cord injury (SCI) is a traumatic neurological disease that causes sensory, motor, and autonomic dysfunction.^1^ Surveys conducted by the World Health Organization have shown that the annual global incidence of SCI is 40–80 cases per million people.^2^ SCI is mainly caused by vertebral body displacement or protrusion of bone fragments into the spinal canal due to traffic accidents, fall injuries, falls, or trauma, resulting in varying degrees of damage to the spinal cord or cauda equina.^3^ Spinal cord injury can cause pathological changes, such as neuronal apoptosis, neuroinflammation, and oxidative stress.^3^ However, the ability of nerves to self‐repair is limited, and the efficacy of various treatments applied to repair nerves after spinal cord injury, such as neuroprotective and neuroregenerative therapies, is unsatisfactory.^4^, ^5^ Therefore, there is an urgent need for further research into effective treatments for SCI.

Previous studies have shown that necrosis and apoptosis play important roles in the progression of SCI.^6^ Upon damage to the blood‐spinal cord barrier, a large number of inflammatory factors are released, which eventually trigger a series of inflammatory responses.^7^ Axonal regeneration after SCI can be regulated by inhibiting the inflammatory response and apoptosis of injured cells, improving SCI.^8^, ^9^ However, the underlying mechanism remains unclear.

The tumor protein p53‐inducible nuclear protein 2 (TP53INP2, also referred to as the diabetes‐ and obesity‐related gene, DOR) is a bifunctional protein that regulates transcription and enhances hunger‐induced autophagy.^10^ TP53INP2 is involved in the occurrence and development of various diseases, such as cancer, myocardial injury, and obesity.^11^, ^12^, ^13^ A recent study indicated that TP53INP2 mRNA expression levels were significantly enhanced in patients with SCI in comparison to those in the control group.^14^ However, the role of TP53INP2 in SCI remains largely unclear.

This study aims to realize and confirm the unknowns in the mechanism of TP53INP2 impact on aSCI mouse model and cellular model.

## 2 MATERIALS AND METHODS

### 2.1 Establishment of the contusion SCI mouse model

Male C57BL/6 mice (20–25 g, 8 weeks old) were purchased from Vital River Laboratory Animal Co., Ltd. Mice were kept in cages with appropriate water and food and kept under standard conditions (25°C ± 1°C and humidity of 50% ± 5%). All animal experiments were approved by the Animal Ethics Committee of the Southeast University (approval number: 2022YHGF223).

Before the experiment, the mice were randomly divided into sham and SCI groups (*n* = 15 per group). The contusion SCI mouse model was constructed according to previously described methods.^15^ Briefly, the mice were intraperitoneally anesthetized with 3% sodium pentobarbital (40 mg/kg; Sigma‐Aldrich). A longitudinal incision was made along the medial dorsal line on the midline of the back to the aponeurotic and muscular planes, thereby exposing the posterior vertebral arches from T8 to T12. Under a dissecting stereomicroscope, a 3 mm long laminectomy was performed, including the caudal end of the T10 vertebral body and the rostral end of the T11 vertebral body. The SCI model was established by tapping the T10 segment of the thoracic spine with a force of 60 kdyn/cm^2^. During the period of recovery from anesthesia, the mice were housed in a temperature‐controlled chamber until thermoregulation was reestablished.

### 2.2 Basso‐Beattie‐Bresnahan (BBB) motor score

The motor abilities of the mice were assessed using the BBB score.^16^ Briefly, mice were placed on a platform and hind limb walking and limb movements were recorded by two observers. The score can be divided into 21 criteria, namely 0–21 points. Double‐blinded observation of the mouse's locomotion was carried out respectively at 1, 7, 14, 21, and 28 days after SCI. The mice were divided into three categories according to the score: a score of 0–7 for the early stage, showing little or no hind limb movement; a score of 8–13 for the intermediate stage, showing intervals of uncoordinated stepping; and a score of 14–21 for the late stage, showing forelimb and hind limb coordination.

### 2.3 Experimental design and animal treatment

To evaluate the effects of TP53INP2 on spinal cord injury, mice were randomly divided into four groups (*n* = 15 per group): sham, SCI, SCI + control siRNA, and SCI + TP53INP2‐siRNA. In the SCI + Control‐siRNA group and SCI + TP53INP2‐siRNA group, 15 min after mice received SCI surgery,^15^ Control‐siRNA (1 μM, sc‐37007; Santa Cruz Biotechnology), or TP53INP2‐siRNA (1 μM, sc‐154565; Santa Cruz Biotechnology) was used to treat the mice via intrathecal injection for 3 days (0, 1, and 2 days). Mice were assessed for motility using the BBB score on days 1, 3, 7, 14, 21, and 28 after SCI, and for spinal cord edema by measuring spinal cord water content on Day 7 after SCI. Seven days after SCI, the mouse spinal cord tissues were collected for subsequent experiments.

### 2.4 Measuring the water content of the spinal cord^15^

After 7 days of treatment, the mouse spinal cord tissue was obtained and weighed (wet weight). Spinal cord tissues were placed at 80°C and weighed (dry weight) after the water had evaporated completely, and the spinal cord water content was calculated according to the formula, spinal cord water content = [(wet weight‐dry weight)/wet weight] × 100%.

### 2.5 Hematoxylin and eosin (H&E) staining

The spinal cord tissues were fixed with 4% paraformaldehyde, dehydrated in an ethanol gradient, embedded in paraffin, and cut into 5‐μm thickness slices. The sections were dewaxed and hydrated, and stained with H&E (Sigma) according to the manufacturer's instructions. The spinal cord tissue morphology was observed under the microscope (Olympus CX21) with a magnification of 400×.

### 2.6 TUNEL assay

Apoptosis of cells in the injured spinal cord was detected using the TUNEL assay.^17^ The spinal cord tissue was fixed with 4% paraformaldehyde for more than 24 h, and 2–3 μm paraffin sections were taken after dehydration. The slices were then dewaxed in xylene for 5–10 min, washed with anhydrous ethanol for 5 min, soaked in 0.2% Triton X‐100 for 15 min, and soaked in DAB solution for 30 min. Images were analyzed using Image‐Pro Plus 6.0 software (Media Cybernetics, Inc.), and the slices were photographed and counted under an optical microscope.

### 2.7 RNA extraction and RT‐qPCR

Total RNA was isolated from the mouse spinal cord tissue and BV‐2 cells using a TRIzol kit (Vazyme) according to the manufacturer's instructions. Extracted total RNA was reverse‐transcribed into cDNA with PrimeScript® RT Master Mix Kit (Takara). Subsequently, RT‐qPCR was performed using AceQ qPCR SYBR Green Master Mix (Vazyme), and the relative mRNA expression of TP53INP2 was calculated with 2^−^^ΔΔCt^.^18^ GAPDH was used as an internal control. Primer sequences for PCR were listed as following:

TP53INP2, forward: 5′‐CCAGCCTTTTCTTCAACACC‐3′;

Reverse 5′‐GCCCCTCTGCAGTAAAACAG−3′;

GAPDH, forward 5′‐CATCATCCCTGCCTCTACTGG−3′;

Reverse 5′‐GTGGGTGTCGCTGTTGAAGTC−3′.

### 2.8 Western blot analysis^19^

Mouse spinal cord tissue and BV‐2 cells were lysed with RIPA buffer (Solarbio) to extract total protein, and the protein concentration was detected using a BCA kit (Beyotime). Equal amounts of protein were separated by 12% SDS‐PAGE and then transferred to PVDF membranes (Millipore). Membranes were then blocked with 5% milk for 1 h and incubated overnight at 4°C with primary antibodies including anti‐rabbit TP53INP2 (DF14312, Affinity Biosciences LTD, dilution rate: 1:1000) anti‐rabbit cleaved‐Caspase3 (9661S; Cell Signaling Technology, dilution rate: 1:1000) and anti‐rabbit GAPDH (5174; Cell Signaling Technology, dilution rate: 1: 1000). The primary antibodies were diluted with 5% skimmed milk. The next day, the membrane was washed three times with TBST buffer. The membranes were then incubated with horseradish peroxidase‐labeled secondary antibodies (AS1107; ASPEN, dilution rate: 1:10,000) for 2 h and visualized with ECL chemiluminescence solution (AS1059; ASPEN) using Kodak medical X‐ray film (XBT‐1; Kodak) in darkroom. AlphaEaseFC software processing system was used to analyze the optical density value of the target band.

### 2.9 Apoptosis detected by flow cytometry^20^

Apoptosis was detected using an Annexin V‐FITC/PI cell apoptosis detection kit (Beyotime), according to the manufacturer's instructions. Briefly, approximately 1 × 10^6^ cells were incubated with 5 μL Annexin V for 15 min at room temperature, followed by 10 μL PI (10 mg/mL) for 5 min at room temperature in darkness. Finally, the samples were analyzed using a flow cytometer (BD Biosciences).

### 2.10 Enzyme‐linked immunosorbent assay (ELISA)^15^

Mouse serum and BV‐2 cell supernatants were collected by centrifugation at 12,000 × *g* for 5 min. The supernatants were assayed for IL‐1β, TNF α, IL 6, and IL‐10 using the corresponding ELISA kits according to the manufacturer's instructions (Thermo Fisher Scientific).

### 2.11 Cell culture and SCI cell model conduction

Mouse microglia BV‐2 were purchased from the American Type Culture Collection (ATCC). BV‐2 cells were cultured in Dulbecco's Modified Eagle's Medium/Ham's F12 (DMEM/F‐12, 1:1; Gibco) medium containing 10% fetal bovine serum (FBS; Gibco). Cells were placed in a 5% CO_2_, 37°C incubator, and the medium was changed every other day. To investigate the molecular mechanism involved in the role of TP53INP2 in secondary SCI‐induced apoptosis and inflammatory response, a SCI cell model using lipopolysaccharide (LPS)‐treated BV‐2 cells was conducted as previously described.^21^ Briefly, BV‐2 cells were cultured with medium containing 100 ng/mL LPS (Solarbio) at 37°C for 24 h to induce cellular damage.

### 2.12 Cell transfection

When cell confluency was approximately 80%, Control‐siRNA or TP53INP2‐siRNA were transfected into BV‐2 cells through Lipofection using a common transfection reagent Lipofectamine™ 2000 (Invitrogen). Transfection efficiency was measured by RT‐qPCR 24 h after transfection.

### 2.13 Statistical analysis

All experiments were repeated at least thrice. Data were analyzed with SPSS 18.0 software (SPSS, Inc.) and presented as mean ± SD. Differences between multiple groups were tested using one‐way analysis of variance followed by Tukey's test. Student's t‐test was used to compare differences between two groups. Statistical significance was set at *p* < .05.

## 3 RESULTS

### 3.1 TP53INP2 is upregulated in the spinal cords of SCI mouse

**Figure 1:**
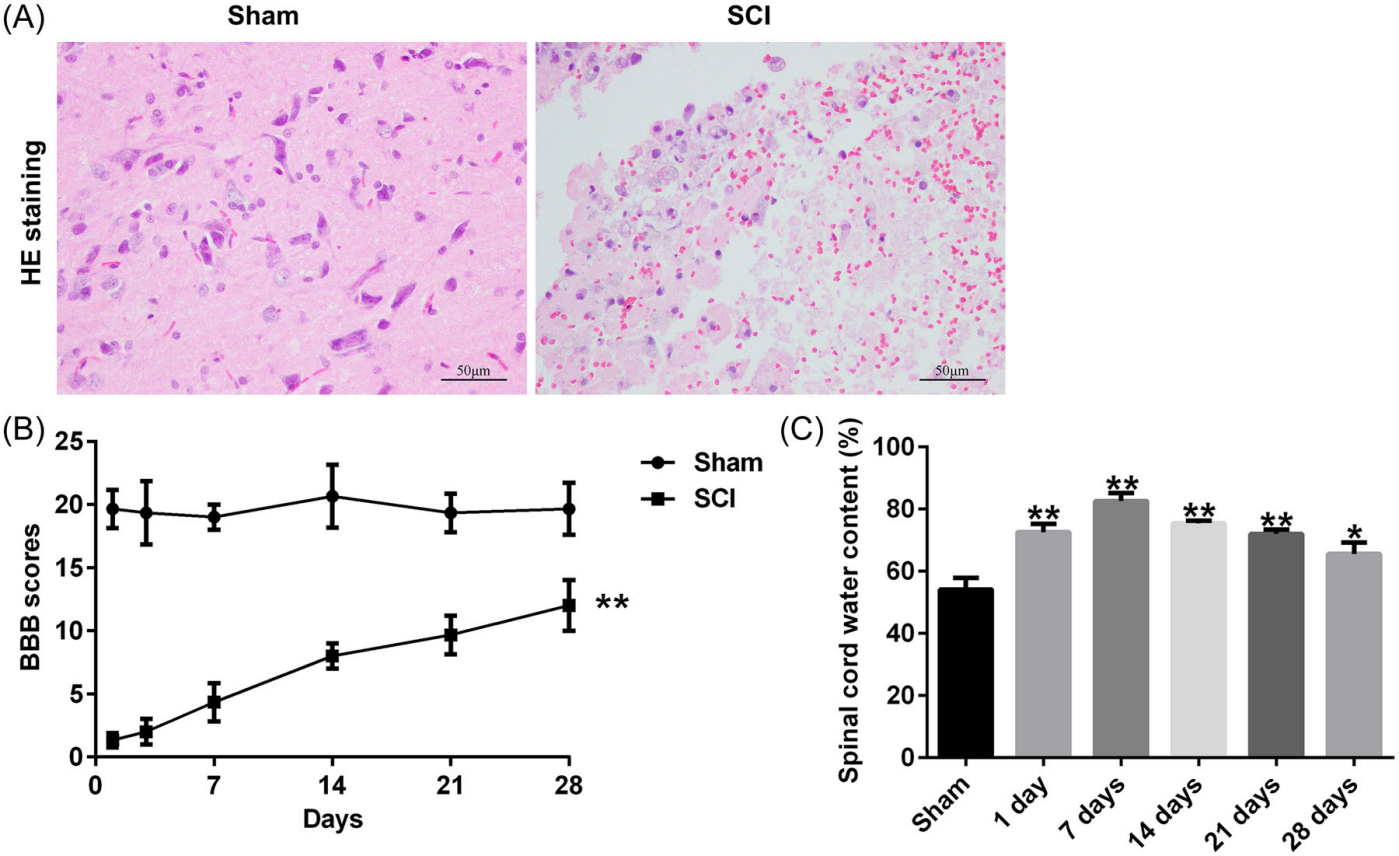
Evaluation of successful spinal cord injury (SCI) mouse model establishment. (A) Histopathological evaluation of spinal cord tissue from each group was detected by H&E staining (bar = 50 μm). (B) Motor function of mice was assessed by BBB scores after SCI. (C) Spinal cord water content measurement (*n* = 6). *, **p < .05, 0.01 versus sham group. BBB, Basso‐Beattie‐Bresnahan; H&E, hematoxylin and eosin.

**Figure 2:**
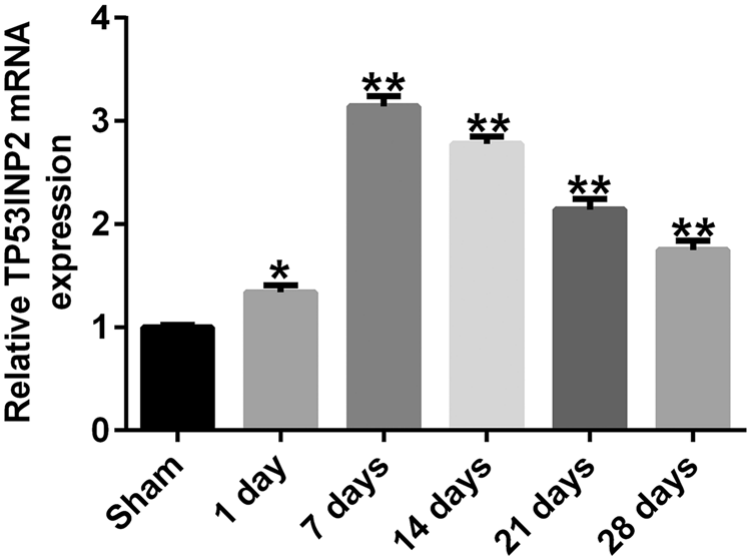
Expression of TP53INP2 in the spinal cord tissue of SCI mouse model. Detecting expression levels of TP53INP2 through RT‐qPCR. *, **p < .05, 0.01 versus sham group. SCI, spinal cord injury.

The function of TP53INP2 in SCI was investigated using a mouse model. Histopathological changes in spinal cord tissues were evaluated by H&E staining, and the results showed that the edema, congestion, and structural damages were observed in the spinal cord of SCI mice (Figure 1A). Motor ability was assessed using BBB scoring at 1, 7, 14, 21, and 28 days after SCI establishment. A spontaneous functional recovery was observed in SCI mice during the experiment, but the BBB scores were significantly lower in the SCI group than in the sham group (Figure 1B), indicating the neurological function reduction in SCI mouse. Moreover, the spinal cord water content of SCI mice was significantly higher than that of the sham group mice and reached its highest level on day 7 after SCI (Figure 1C). As shown in Figure 2, the expression of TP53INP2 was significantly increased in the spinal cord tissue of the SCI group. These results implied that TP53INP2 may be involved in the development of SCI.

### 3.2 TP53INP2 knockdown alleviates spinal cord injury

**Figure 3:**
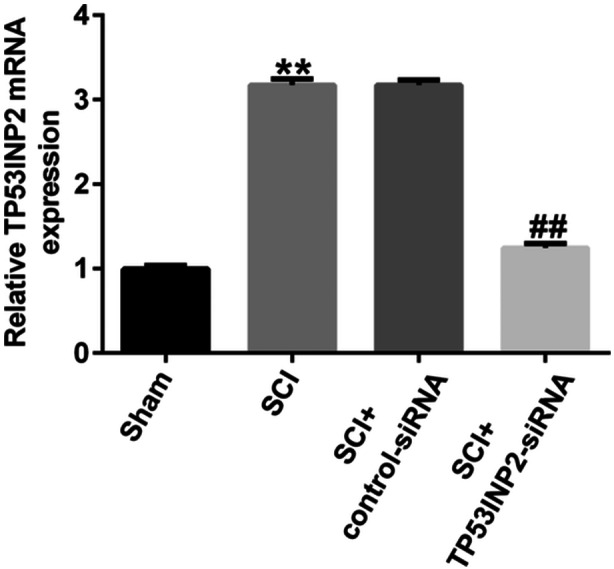
TP53INP2 knockdown in spinal cord injury (SCI) mice. Detecting the mRNA levels of TP53INP2 through RT‐qPCR. **p < .01 versus Sham; ^##^ *p* < .01 versus SCI+Control‐siRNA.

**Figure 4:**
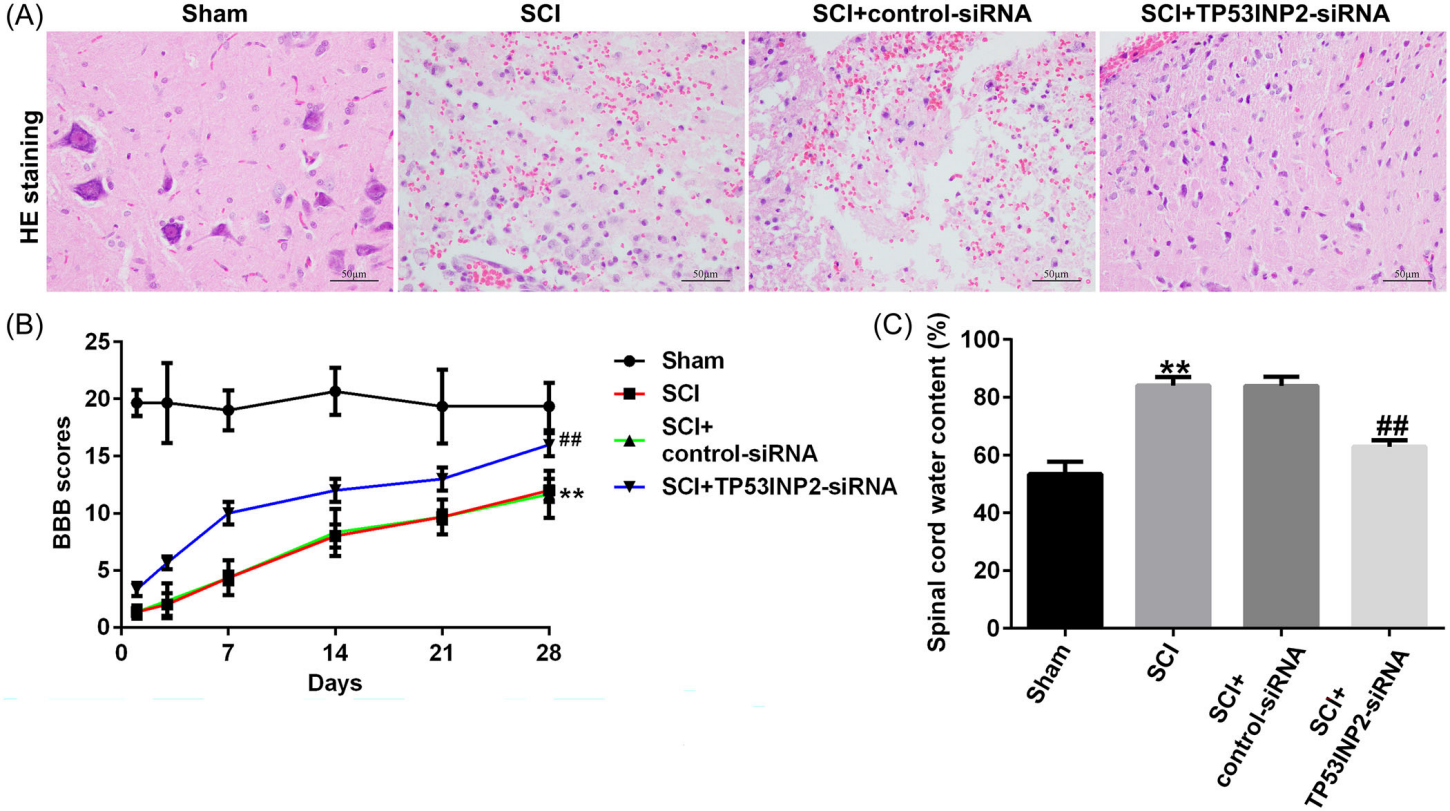
TP53INP2 knockdown alleviates symptoms in spinal cord injury (SCI) mice. (A) Histopathological evaluation of spinal cord tissue from each group was detected by H&E staining (bar = 50 μm). (B) BBB scores were used to evaluate the motor function. (C) Measurement of spinal cord water content (*n* = 6). **p < .01 versus Sham; ^##^ *p* < .01 versus SCI+Control‐siRNA. BBB, Basso‐Beattie‐Bresnahan; H&E, hematoxylin and eosin.

To explore the role of TP53INP2 in SCI, we injected control siRNA or TP53INP2‐siRNA into the sheaths of mice subjected to SCI surgery. The mice were subsequently assessed for locomotion and TP53INP2 expression. The RT‐qPCR results showed that TP53INP2‐siRNA injection into SCI mice significantly decreased TP53INP2 levels (Figure 3). The results of H&E staining showed that the edema, congestion, and structural damages in the spinal cord of SCI mice were attenuated after TP53INP2‐siRNA treatment (Figure 4A). The BBB score was significantly lower in the SCI group than in the sham group (Figure 4B), and spinal cord water content was significantly higher in the SCI group (Figure 4C). In SCI mice, the knockdown of TP53INP2 significantly increased the BBB score and decreased the water content in the spinal cord (Figure 4B,C). These results suggested that TP53INP2 knockdown alleviated SCI.

### 3.3 TP53INP2 knockdown suppresses apoptosis and inflammatory responses in spinal cord injury

**Figure 5:**
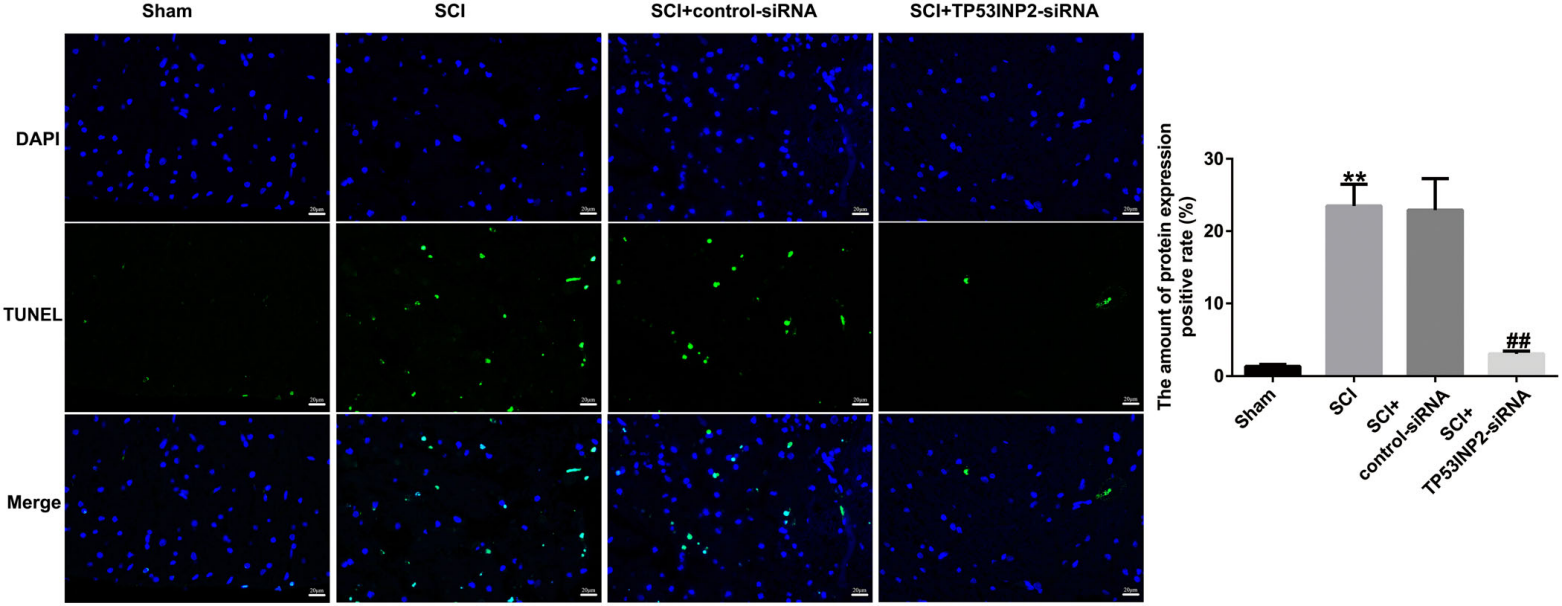
TP53INP2 knockdown suppresses neuronal apoptosis in spinal cord injury (SCI) mice. TUNEL assay was performed to determine neuronal apoptosis in the spinal cord tissue of mice. **p < .01 versus Sham; ^##^ *p* < .01 versus SCI+Control‐siRNA.

**Figure 6:**
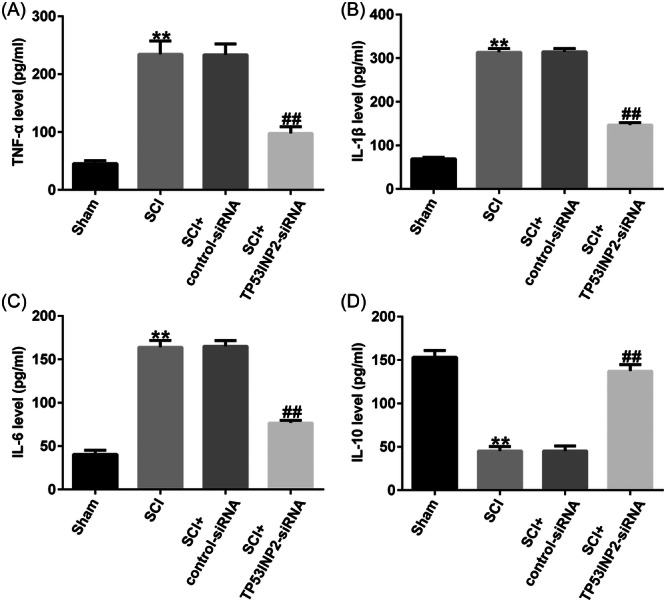
TP53INP2 knockdown suppresses inflammatory response in spinal cord injury (SCI) mice. ELISA was used to measure the levels of (A) TNF‐α, (B) IL‐1β, (C) IL‐6, and (D) IL‐10. **p < .01 versus sham; ^##^ *p* < .01 versus SCI+Control‐siRNA.

The role of TP53INP2 in apoptosis and inflammatory responses was detected using TUNEL and ELISA. The results revealed that cell apoptosis was significantly enhanced in SCI mice (Figure 5). Additionally, the concentrations of TNF‐α, IL‐1β, and IL6 in spinal cord tissues were significantly higher, and the secretion of IL‐10 was significantly lower in the SCI group (Figure 6A–D). In SCI mice, when TP53INP2 was downregulated, the apoptosis of neurons was significantly reduced in spinal cord tissue (Figure 5), and TNF‐α, IL‐1β, and IL 6 were significantly reduced, while IL‐10 secretion was significantly increased (Figure 6A–D). These results suggested that TP53INP2 knockdown suppressed inflammatory responses and apoptosis in spinal cord injury.

### 3.4 TP53INP2 is upregulated in LPS‐treated BV‐2 cells

**Figure 7:**
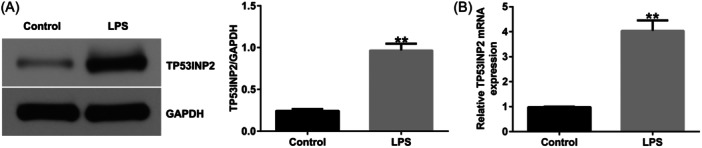
Expression of TP53INP2 in LPS‐induced BV‐2 cells. (A) Detecting the protein levels of TP53INP2 through western blot assay. (B) The levels of TP53INP2 in groups were detected with RT‐qPCR. **p < .01 versus Control. LPS, lipopolysaccharide.

To further investigate the effects of TP53INP2 in spinal cord injury, BV‐2 cells were treated with LPS to establish an SCI cell model. The expression of TP53INP2 was significantly increased in LPS‐treated BV‐2 cells (Figure 7A,B).

### 3.5 TP53INP2 knockdown inhibits inflammation and apoptosis in LPS‐induced BV‐2 cells

**Figure 8:**
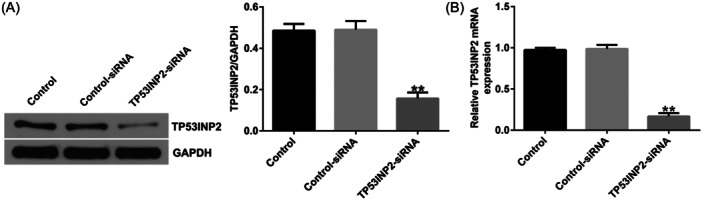
TP53INP2‐siRNA significantly reduced TP53INP2 expression in BV‐2 cells. (A) Detecting the protein levels of TP53INP2 in BV‐2 cells transfected with Control‐siRNA or TP53INP2‐siRNA through western blot assay. (B) The levels of TP53INP2 in BV‐2 cells transfected with Control‐siRNA or TP53INP2‐siRNAs were detected with RT‐qPCR. **p < .01 versus Control‐siRNA.

**Figure 9:**
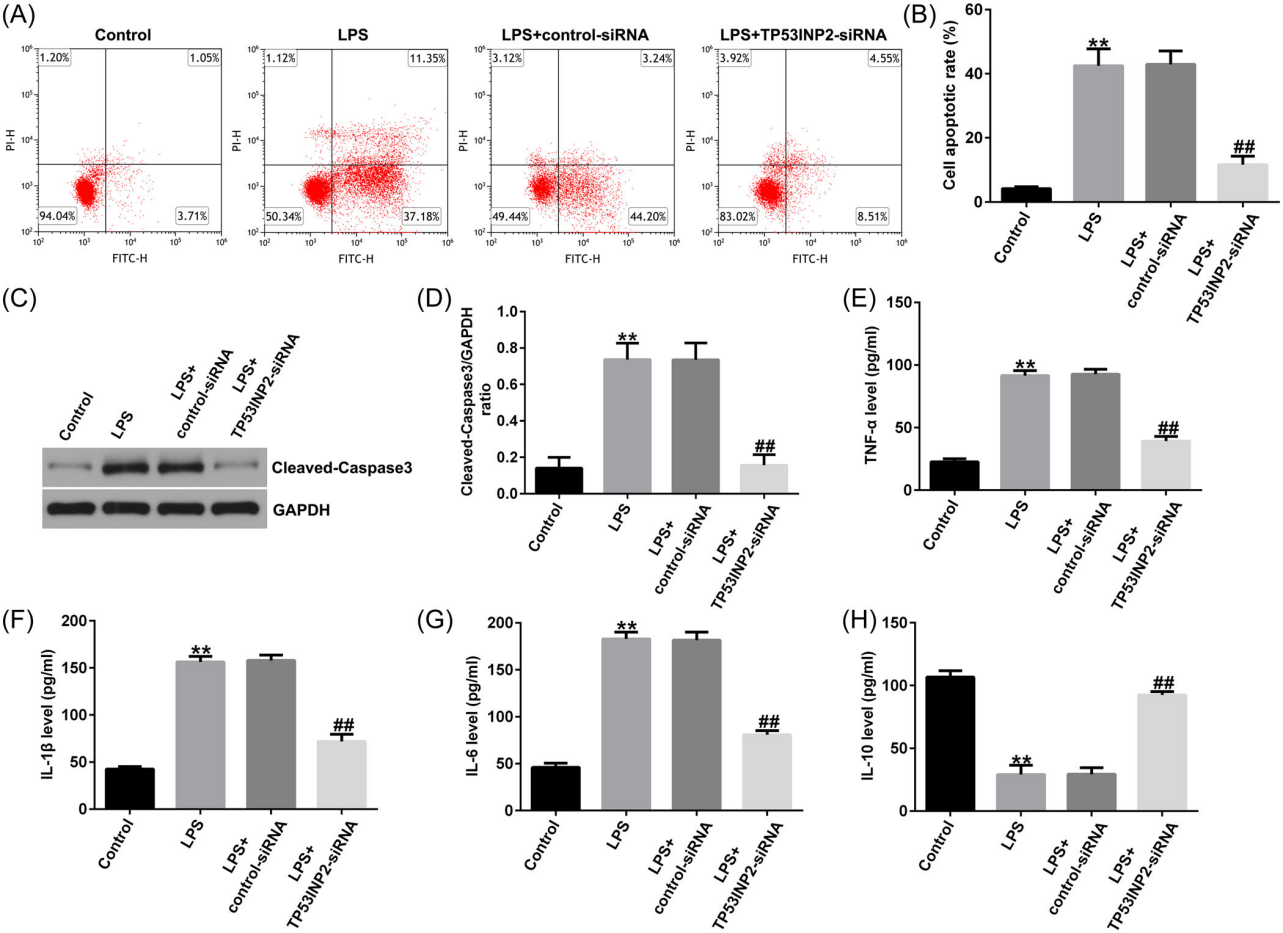
TP53INP2 knockdown inhibits apoptosis and inflammation in lipopolysaccharide (LPS)‐induced BV‐2 cells. (A, B) Detection of cell apoptosis by flow cytometry. (C) Detecting protein levels of cleaved‐Caspase3 in BV‐2 cells with western blot assay. (D) Cleaved‐Caspase3/GAPDH ratio. (E–H) Detection of inflammatory factors in supernatant of BV‐2 cells with ELISA. **p < .01 versus Control; ^##^ *p* < .01 versus LPS+Control‐siRNA.

To explore the effects of TNP53INP2 on spinal cord injury, we transfected Control‐siRNA or TP53INP2‐siRNA into BV‐2 cells for 4 h and then treated the cells with LPS for 24 h. First, we confirmed that compared to the control siRNA group, TP53INP2‐siRNA significantly reduced TP53INP2 in BV‐2 cells (Figure 8A,B). Analysis by flow cytometry showed that the apoptosis of BV‐2 cells significantly increased after LPS induction, whereas TP53INP2 knockdown significantly reduced apoptosis in LPS‐induced BV‐2 cells (Figure 9A,B). In addition, LPS induction significantly increased the levels of cleaved‐Caspase3 and the cleaved‐Caspase3/GAPDH ratio in BV‐2 cells, while TP53INP2 knockdown significantly decreased the cleaved‐Caspase3 and cleaved‐Caspase3/GAPDH ratios (Figure 9C,D). Inflammatory factors in BV‐2 cells were detected by ELISA. The results showed that LPS increased the levels of TNF‐α, IL‐1β, and IL‐6 and decreased the release of IL‐10 compared to the control group (Figure 9E–H). However, TP53INP2 knockdown significantly decreased the secretion of TNF‐α, IL‐1β, and IL‐6, and enhanced the secretion of IL‐10 (Figure 9E–H).

## 4 DISCUSSION

SCI is difficult to treat clinically, and the underlying mechanisms are not fully understood.^1^, ^22^ Currently, several approaches have been applied to the treatment of SCI, including surgical and pharmacological treatments; however, their efficacy is unsatisfactory.^4^ Inhibiting cellular inflammation and apoptosis can effectively alleviate SCI; however, effective factors in the control of secondary damage following SCI require further exploration.

TP53INP2 has been reported to be involved in various diseases.^11^, ^12^, ^13^, ^23^, ^24^, ^25^ A recent study indicated that TP53INP2 was significantly upregulated in patients with SCI in comparison to those in the control group.^14^ However, the role and mechanism of action of TP53INP2 in SCI remains unclear. In the present study, we investigated the specific role and mechanism of action of TP53INP2 in SCI. SCI mouse model was constructed according to previously described methods.^15^ And consistent with the previous study,^15^ SCI induction significantly induced the edema, congestion, and structural damages of the spinal cord tissue of mice, reduced the BBB score, and enhanced the spinal cord water content of the mice, indicating the successful establishment of SCI mouse model. LPS induced BV‐2 cells have been widely used in SCI in vitro studies.^21^, ^26^, ^27^ In this study, SCI in vitro model was established by treating BV‐2 cells with LPS, and the data indicated that LPS significantly induces apoptosis and inflammatory response in BV‐2 cells, indicating the successful establishment of the model.^21^, ^28^, ^29^ However, on the contrary, studies have reported that low‐dose LPS suppresses PC‐12 cell apoptosis, thus reducing SCI damage,^30^ which requires further in‐depth investigation. Using the in vivo SCI mouse model and in vitro SCI cell model, we found that TP53INP2 was significantly enhanced in the SCI mouse spinal cord tissue and LPS‐induced BV‐2 cells. We confirmed that TP53INP2 knockdown alleviated spinal cord injury in mice, as evidenced by an increased BBB score, reduced water content in the spinal cord.

Secondary injury is crucial in the pathological progression of SCI and has a significant effect on neuronal apoptosis and inflammatory responses, which can lead to severe nerve damage and dysfunction.^28^, ^29^ Inhibiting the activation of microglia in the damaged area and subsequent inflammatory response, as well as neuronal apoptosis, is a key therapeutic strategy for the rehabilitation of SCI patients.^7^, ^28^, ^31^ Therefore, we explored the role of TP53INP2 in neuronal apoptosis and the inflammatory response. The current study demonstrated that TP53INP2 knockdown significantly inhibited the inflammatory response and apoptosis in SCI mice and LPS‐induced BV‐2 cells.

There were also some limitations of this study. First, in this study, we used the dry mass comparison method to evaluate moisture in the spinal cord, while the Karl Fisher method was more accurate in measuring moisture in the spinal cord. Besides, mouse microglia BV‐2 was used to conduct SCI cell model, while using a neural model cell or neurolastoma to study the effect of TP53INP2 on neuronal damage in SCI would make the research more substantial and convincing. We will further study these issues in our next research in the future.

In conclusion, this study indicated that TP53INP2 knockdown plays a protective role in SCI by suppressing inflammatory responses and neuronal apoptosis. These findings provide new targets and directions for the diagnosis and treatment of SCI.

## AUTHOR CONTRIBUTIONS

**Penghao Sun**: Conceptualization; data curation; investigation; writing—original draft; writing—review & editing. **Jinchuan Chen**: Methodology; resources; software. **Rujie Qin**: Formal analysis; supervision; visualization; writing—review & editing.

## CONFLICT OF INTEREST STATEMENT

The authors declare no conflicts of interest.

## ETHICS STATEMENT

All animal experiments were approved by the Animal Ethics Committee of the Southeast University (approval number: 2022YHGF223).

## Data Availability

The datasets used and/or analyzed during the current study are available from the corresponding author upon reasonable request.
